# Herd-level risk factors for cow and calf on-farm mortality in Estonian dairy herds

**DOI:** 10.1186/s13028-020-0513-x

**Published:** 2020-03-12

**Authors:** Kaari Reimus, Karin Alvåsen, Ulf Emanuelson, Arvo Viltrop, Kerli Mõtus

**Affiliations:** 1 Institute of Veterinary Medicine and Animal Sciences, Estonian University of Life Science, 51014 Tartu, Estonia; 2 Department of Clinical Sciences, Swedish University of Agricultural Sciences, 750 07 Uppsala, Sweden

**Keywords:** Dairy cattle, Euthanasia, Mortality, Unassisted death, Housing conditions

## Abstract

**Background:**

On-farm mortality (unassisted death and euthanasia) is the unwanted loss of animals, and it comes with negative economic consequences. On-farm mortality rates reflect a herd’s animal welfare status. The objective of this historical longitudinal single cohort study was to identify the associations between herd characteristics, animal housing conditions and management routines and within-herd calf and cow mortality rates in participating Estonian dairy herds. All farmers enrolled in the voluntary production recording system with a herd size of 20 or greater cow-years in 2015–2017 were contacted by mail or telephone between October 2017 and March 2018. The survey included questions about management routines and housing conditions of calves up to 3 months of age and of cows. In total, 214 completed questionnaires were returned, corresponding to a 63.3% response rate. The within-herd mortality rate of calves (aged 21–90 days) and cows (cattle over 24 months of age) in years 2017–2018 were calculated and used as outcome variables. Negative binomial and linear regression models were applied for risk factor analysis in calf and cow datasets, respectively.

**Results:**

The median within-herd mortality rate for calves aged 21 to 90 days was 0.15 per 100 calf-months (quartiles 0.00; 0.36). The median within-herd mortality rate for cattle over 24 months of age was 4.57 per 100 cow-years (quartiles 2.44; 6.86). Factors significantly associated with increased mortality of calves were larger herd size, higher proportion of stillbirths and abortions in a herd, prophylactic administration of vitamins to all calves and housing pre-weaned calves in single pens only compared with housing in both single and group pens. Also, farmers who attended more frequent trainings had higher calf mortality rates. Calving in a group pen or in a tie-stall compared with calving in multiple systems was associated with higher calf mortality rates. Higher cow mortality rates were present in herds that had a higher proportion of stillbirths and on farms where employees handled cows. Housing cows in free-stall barns, grazing cows and more frequent hoof trimming were protective for cow on-farm mortality.

**Conclusions:**

This study identified the importance of housing conditions for on-farm cow and calf mortality rates. Our study results suggest that providing good care might ensure better health and welfare of dairy cows and calves.

## Background

On-farm mortality (unassisted death and euthanasia) is an unexpected and undesirable loss of an animal. Negative economic consequences resulting from the death of a cow include loss of production (including potential lifetime milk yield, meat and future offspring), possible treatment expenditures, and cost of waste management in addition to indirect costs (e.g., investments in labour, housing, feeds and veterinary expenses). On dairy farms, heifers are reared mostly for replacement [1]. Therefore, the death of young stock deteriorates the future potential of a dairy herd due to delayed genetic progress, lower chances for voluntary culling of lactating cows, increased cost of replacement and limited possibilities to earn income from the sale of surplus heifers [2]. Increased on-farm mortality is associated with deteriorated animal health and welfare [3, 4] and, therefore, is an ethical issue of public concern. It has been shown that herds with high calf mortality use antimicrobials more frequently [5], and there is an association between antimicrobial resistance and calf mortality [6]. A trend toward increased dairy cattle mortality has been reported in several countries [7–10]. Although there are no studies evaluating the long-term trends of dairy cattle mortality in Estonia, recent data indicate that on-farm mortality rates (MRs) in the Estonian dairy cattle population have increased. The overall on-farm MR of Estonian dairy cattle was 6.95 [95% confidence interval (CI) 6.87; 7.04] per 100 animal-years during 2013 and 2014 [11], whereas the MR in 2018 was 7.59 (95% CI 7.45; 7.72) per 100 animal-years [12].

Due to an increased global demand for milk products and need to optimise costs of production, there is a pressure toward intensification of dairy farm operations [13]. In 2017, there were 86,409 dairy cows in Estonia kept in 1566 herds. Of these herds, 84.1% had a herd size of fewer than 50 cows but constituted only 9.3% of the total Estonian dairy cow population. Although the median herd size of Estonian dairy herds is relatively small, 85.2% of the dairy cows were kept in herds with more than 100 cows [12]. In general, Estonian dairy cows are high yielding: the national average milk yield per cow per year has been over 9000 kg during the last 3 years [14], taking the second place in the European Union [15].

Previous studies have identified herd-level risk factors related to dairy cow on-farm mortality. Thus, larger herd size, increased percentage of stillbirths, higher somatic cell count, increased herd calving interval, lower herd milk yield, no summer grazing, feeding total mixed ration, increased proportion of purchased cows, herd not vaccinated for endemic bovine infectious diseases, increased proportions of dairy cows with clinical mastitis and infertility problems as well as increased incidence of lameness and injuries among removed cows are examples of factors shown to be associated with dairy cow on-farm mortality [9, 16–18]. Unfortunately, not many studies have aimed to analyse how factors related to cows’ daily routines and environment are associated with on-farm cow mortality. Still, it has been confirmed that management and housing conditions of cows influence their health and well-being as reflected in on-farm mortality [19].

Several studies have investigated herd-level factors associated with calf mortality. Some examples of the factors identified as important in the context of calf mortality are herd size, characteristics of the calving pen or area, calf housing system and group size, shorter milk feeding period, length of period a calf spends in a calving pen, approach to bovine herpesvirus 1 and bovine viral diarrhoea virus control as well as farmers’ mindset [20–24]. Still, parallel to the intensification of the dairy production, calf-rearing practices have probably changed and, thus, more up-to-date studies focusing on finding risk factors associated with calf management and rearing conditions to mortality are needed.

Numerous studies have arrived at the conclusion that on-farm mortality has a high between-herd variation [11, 25, 26], indicating that dairy cattle health and welfare vary heavily across herds and it would therefore be worthwhile to analyse the differences in their management systems. Identifying herd-level risk factors for on-farm mortality is highly relevant for many stakeholders such as farmers, veterinary advisors and authorities, and of great value especially for larger herds where intervention can positively affect a larger number of animals. As shown in previous studies, the two most vulnerable groups of cattle in terms of mortality are young calves and adult cows [11, 27, 28]. Therefore, the aim of the present study was to determine herd characteristics and management routines associated with dairy calf and cow on-farm mortality.

## Methods

### Study population

The study population included all Estonian dairy herds that had 20 or greater cow-years from 2015 to 2017 and that participated in the voluntary milk recording system. The list of herds that met the herd size criteria (n = 338) was retrieved from the Estonian Livestock Performance Recording Ltd. (national milk recording centre including ~ 95% of all dairy cows in Estonia, hereafter called the “milk recording register”).

### Questionnaires

A questionnaire was developed to collect data about herd characteristics, animal housing and management routines that were hypothesised to be associated with dairy calf and cow on-farm mortality. The questionnaire consisted of three parts. The first part included questions about the farm in general (name and identification number of the farm, respondent’s name, position and years of working experience with dairy cattle). The respondents were asked to specify the identification number of the farm to which the given answers were related in case the farm had more than one farm unit. The second part of the questionnaire included questions about cow management, housing conditions and calving management. The third part included questions about housing conditions and management routines related to calves up to 3 months of age. The respondents were asked to specify any large changes in animal housing conditions or management routines during the last 4 years. Altogether, the questionnaire included 46 multiple-choice questions and 3 open-ended questions. The questionnaire was pre-tested in three dairy farms to identify any questions that might be misunderstood or lacking any answer categories, before it was distributed.

The questionnaires were mailed to the 338 farmers in October 2017 by the milk recording register, together with the monthly milk test results. The questionnaire had a cover letter that introduced the aim of the study and specified that the person eligible for responding was the owner of the farm, the manager or another person involved with the daily handling of animals. The farm managers were also informed about their anonymity during the data processing and presentation of results. A pre-stamped and addressed envelope was also included to facilitate the return of the completed questionnaire. A reminder postcard was sent to all farmers in December 2017. The farms that had not returned the questionnaire were contacted by phone between February and March 2018. Two pre-trained persons, whose interviewing technique had been standardised, conducted all phone interviews, strictly following the format of the printed questionnaire so that there was a similar understanding of the questionnaire by those who responded by mail and those who answered the questions by phone.

### Data editing

Answers to the postal questionnaires and the questionnaires completed by phone interview were entered to the online survey tool Connect (https://en.connect.ee/). All the answers were then exported from the online survey tool to Excel format. If some response categories had a low number of answers, they were re-categorised or merged into meaningful categories when possible.

Herd size and herd performance data (herd milk yield averages, milk fat/protein ratio, milk somatic cell count, milk urea level, age at first calving, calving interval, length of dry period, calving to first insemination and calving to conception interval, herd average number of inseminations per conception, first insemination conception rate, proportion of stillbirths and abortions, herd number of lactations and age at first insemination) were retrieved from the milk recording register for years 2015 to 2017. Three-year averages were calculated for these data to lower the impact of exceptional years and were used as continuous variables in the statistical analyses. Animal register data were used to calculate the within-herd MRs. Due to the possibility that one farm might have more than one animal keeping unit/facility, the animal records were extracted for the farm unit stated by the respondent. This ensured that the calculated MRs could be associated with the questionnaire data of the identical farm unit.

To calculate herd-level MRs for cows and calves, animal-level data from the selected herds was extracted from the Estonian Agricultural Registers and Information Board (government agency responsible for animal data collection in Estonia, hereafter referred to as “Animal Registry”) database for the years 2017 and 2018. Farmers are obliged by law to report the movements and exits (death on farm, euthanasia, slaughter, disappearance, selling) of cattle to the Animal Registry within 7 days [29]. Farmers have to ear tag calves within 20 days after birth [29]. Registry data might therefore miss deaths that occur during the first 3 weeks of age, since farmers could report calves not yet ear tagged as stillborn. The MR for young calves was therefore calculated for calves aged 21 to 90 days to remove possible bias resulting from unreported calf deaths and make risk factor analysis sounder. A “cow” is generally defined as a female dairy animal that has calved at least once, but the date of first calving was not available in the data. The definition of a “cow” in the present study was therefore a female animal of 24 months or older, which is roughly the lowest quartile of the age at first calving of Estonian dairy cows [30]. For calculating the MR, the numerator included the number of deaths (unassisted death and euthanasia) and the denominator included the number of cow-days in the farm unit belonging to the respective age group. The observation period for individual animals started on January 1, 2017 for animals that were in the farm unit at that date and met the age class requirements (age 21–90 days and at least 24 months in calf and cow datasets, respectively). The dataset continuously recruited all observations that qualified to be included in the respective animal classes during the study period. The observation period for imported animals started at the date of import. The contribution of animal risk-time was accounted until mortality (unassisted death or euthanasia), censoring (slaughter, selling, exporting, animal lost or for calves since the age of 90 days) or until the end of the study period.

### Statistical analyses

**Fig. 1:**
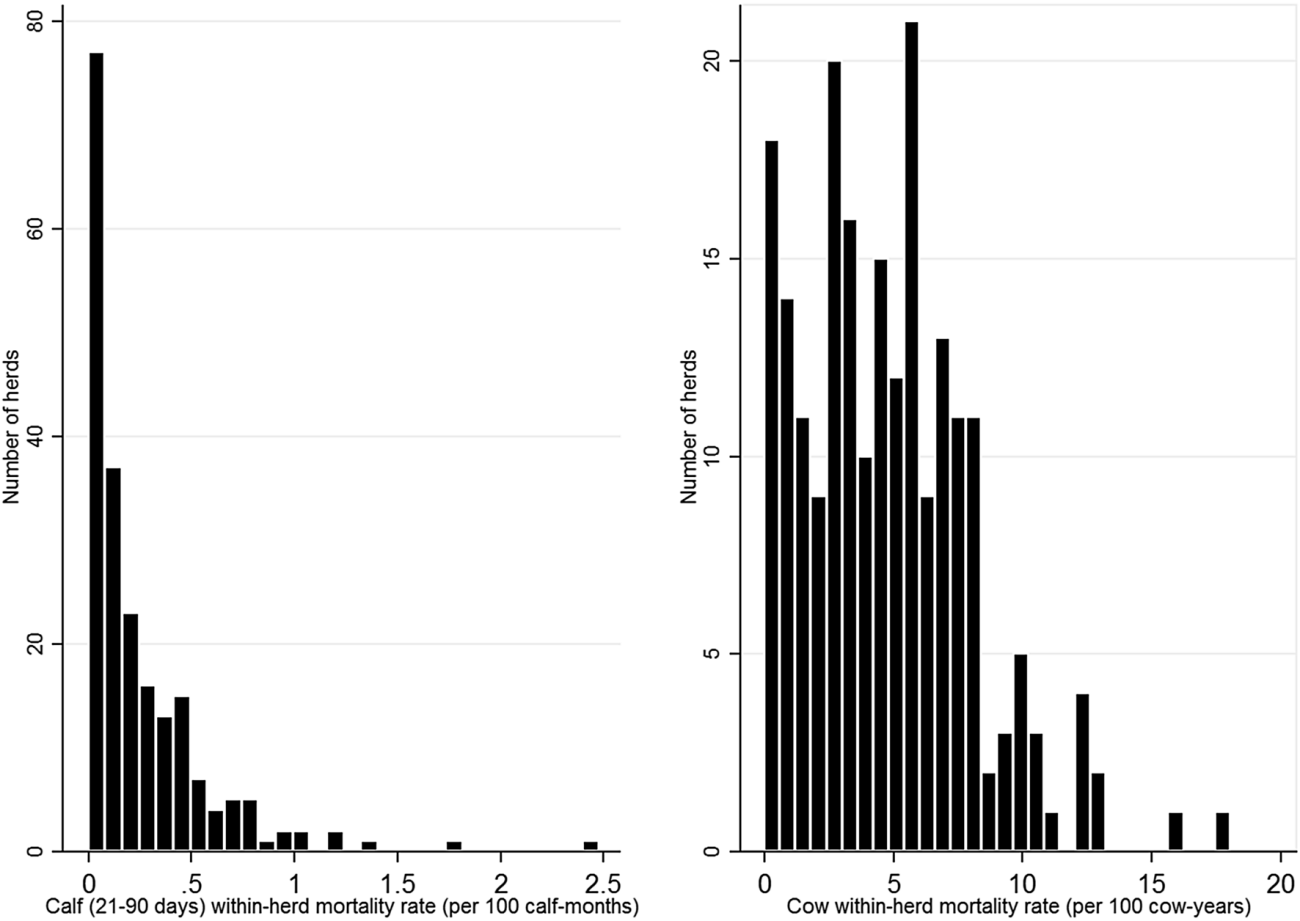
Distribution of within-herd calf (21–90 days of age) and cow mortality rates over years 2017 to 2018 in 212 responding herds in Estonia

The statistical analyses identifying risk factors for mortality were conducted separately for calves and cows. Based on the distribution of the MRs over the years of 2017 and 2018 (Fig. 1), negative binomial regression analysis (‘*nbreg*’ command in Stata ®) and linear regression analysis (‘*reg*’) were used for modelling risk factors in calves and cows, respectively. In the negative binomial model, the number of death events over the years between 2017 and 2018 was the outcome variable, and the total number of calf-months during the respective 2-year period was the exposure that was specified as the “exp” option. In the linear regression model, the outcome variable was the average MR of cattle over 24 months of age between 2017 and 2018. To meet the assumption of normality of the residuals of the linear regression model, a square root transformation was applied for the average cow MR over the years of 2017 and 2018. The overall principle of model building was common for both models. At first, a causal diagram was created to identify a causal pathway between variables and detect possible confounders. According to that, herd size, type of production, housing system, milking system and region were considered as possible confounders. Since the majority of the explanatory variables were plausibly and statistically confounded by herd size, we included “number of cows” to the models when screening for unconditional associations between predictor and outcome variables. A threshold p-value of 0.2 was chosen for detecting potential risk factors to be included into the multivariable models. After that, collinearity between the predictor variables was assessed with a variance inflation factor, where a value larger than 10 was considered to indicate a significant collinearity between predictor variables [31]. No significant collinearity was detected between variables considered as candidates for the multivariable model. Manual backward elimination was used to exclude statistically non-significant (p > 0.05) variables from the multivariable model. To detect confounding effects of the variables, change of the magnitude of the regression coefficients was observed after removing the variable. A change in the regression coefficients of greater than 20% in any of the remaining variables in the model indicated a confounding effect, and the variable was retained in the model [31]. In the model for dairy cows, ‘herd size’ and ‘housing system’ had a confounding effect. Linear associations between continuous predictor and outcome variables were tested by adding a centred linear and centred square term to the model. In case the square term was non-significant, the association between the continuous predictor variable and the outcome was considered linear. In the multivariable models, ‘way of responding to questionnaire’ was included to account for its possible effect. This variable was, however, not included in the final multivariable models due to lack of effect on the regression coefficients. Biologically plausible interactions between variables were tested by adding an interaction term between two variables. Also, interactions were tested between the variable “herd size” (dichotomised at the median value of herd number of cows) and all other predictors in the model. In the cow model, two interactions (“herd size” and “handling animals” as well as “herd size” and “herd average proportion of stillbirths”) were significant. As the model with interaction between “herd size” and “herd average proportion of stillbirths” had lower Akaike information criterion (AIC) and Bayesian information criterion (BIC) values, it was considered better and is presented as the final model. To analyse pairwise associations between all categories of the variables, the reference category was changed. A “margins” command applying a square transformation in response option “expression” was used to obtain predicted mean values of the model estimates of the linear regression model with square root transformed outcome variable.

Diagnostics of the negative binomial regression model included checking for overdispersion, as well as identifying observations with poor fit and high influence. Distribution of the residuals of the final multivariable linear regression model was checked graphically. No problems in overall fit of the models were identified.

Representativeness of the whole population of the study herds in terms of herd size and county was assessed by using descriptive statistics. Statistical analyses were performed with Stata MP14 (StataCorp, College Station, TX, USA).

## Results

### Study herds

**Fig. 2:**
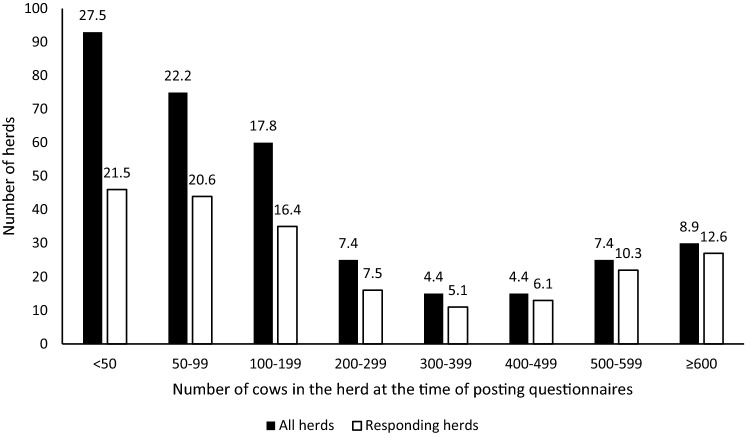
Distribution of all dairy herds in Estonia with more than 20 cows (n = 338) and responding herds (n = 214) based on herd size (numbers on the top of the bars represent the proportion of herds (%) in that herd size category)

**Fig. 3:**
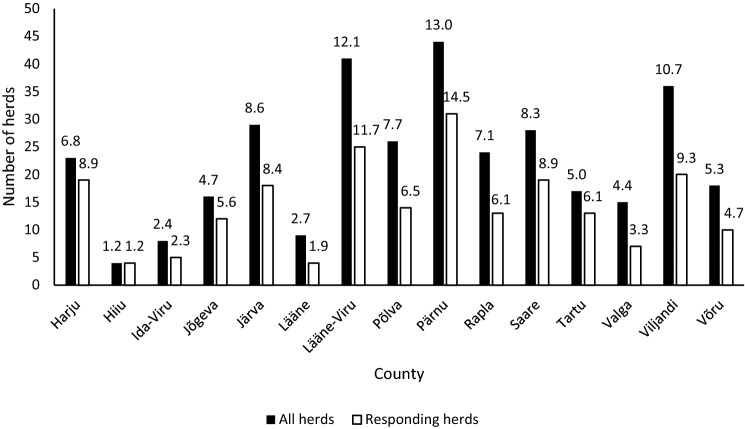
Distribution of all dairy herds in Estonia with more than 20 cows (n = 339) and responding herds (n = 214) based on the location (numbers on the top of bars represent the proportion (%) of herds in that county, Northeast Estonia: Ida-Viru, Lääne-Viru, Jõgeva, Järva county; Southeast Estonia: Tartu, Valga, Võru, Põlva county; Southwest Estonia: Pärnu, Viljandi, Saare county; Northwest Estonia: Harju, Rapla, Lääne, Hiiu county)

By the end of April 2018, a total of 214 responses were received (127 by mail and 87 by calling). Farms that changed production from dairy to beef in 2018 were not considered eligible in the analyses. Due to that, two farms were excluded from the analysis. The final datasets for calves and cows included 212 farms (usable response rate, 62.7%). In total, 145,920 and 148,323 individual calf and 79,767 and 80,691 cow records were used to calculate the on-farm MR for the study farms in years 2017 and 2018, respectively. The smallest herd size category (herds with < 50 cows) was somewhat underrepresented in this study (Fig. 2). The distribution of responding herds based on location (on a county level) compared to all dairy herds in the study population was similar (Fig. 3).

The distribution of MRs for calves aged 21 to 90 days and cows for years 2017–2018 are provided in Fig. 1. The median of the MR for calves aged 21 to 90 days was 0.15 per 100 calf-months (quartile (Q) 1 = 0.00 and Q3 = 0.36). Among the study herds, 66 (31.1%) of the farms had no mortality events among calves aged 21 to 90 days occurring over the study period, and in 7 farms the MR was higher than 1 death per 100 calf-months. The median of the MR for cows was 4.57 per 100 cow-years (Q1 = 2.44 and Q3 = 6.86) (Fig. 1).

### Risk factors associated with calf mortality rate

All variables that were tested as risk factors for calf mortality are presented in Additional files 1 and 2 for categorical and continuous variables, respectively, providing descriptive statistics and unconditional associations with herd on-farm MRs. Variables tested in the multivariable negative binomial regression model are presented in Table 1.

**Table 1:** Descriptive statistics and unconditional associations of variables selected as candidates for multivariable model for within-herd calf (aged 21–90 days) mortality rate in years 2017–2018 in 212 Estonian dairy herds estimated in negative binomial regression model

Variable	Continuous	Categorical	n	Within-herd MR^a^	p-value^b^
	Median (Q1; Q3)	Categories			
Herd size (number of cows)	129.7 (53.7; 459.7)		212		0.015
Herd average milk yield per cow per year (kg)	8787 (6950; 9758)		212		0.132
Herd proportion of stillbirths (%)	7.5 (5.6; 9.5)		212		0.018
Herd proportion of abortions (%)	0.8 (0; 1.6)		212		< 0.001
Herd type^c^		Dairy herd	199	0.26	0.035
		Mixed herd	13	0.09	
Proportion of Holstein breed cows		< 90%	89	0.20	0.160
		> 90%	119	0.28	
		Missing	4	0.35	
Region^d^		Northeast	59	0.29	0.402
		Southeast	44	0.19	
		Southwest	71	0.26	
		Northwest	38	0.22	
Farmer attending trainings within the last 4 years		Once	49	0.15	0.002
		2–3 times	108	0.33	
		More than 3 times	31	0.22	
		No	24	0.14	
Place of calving		Group pen	69	0.32	0.024
		Individual pen	41	0.26	
		Tiestall	84	0.21	
		Combined or other^f^	16	0.07	
		Missing	2	NE^e^	
Time of feeding the first colostrum (hours after birth)		0.5	22	0.19	0.149
		1	87	0.23	
		2	69	0.33	
		3	17	0.19	
		≥ 4	12	0.14	
		Calf drinks by itself	3	NE^e^	
		As soon as possible	2	NE^e^	
Disinfecting navel cord		No	68	0.21	0.070
		Sometimes	51	0.17	
		Always (in more than 95% of calves)	93	0.32	
Prophylactic administration of vitamins to calves		No	64	0.22	0.101
		Sometimes	70	0.21	
		Yes (in more than 95% of calves)	78	0.31	
Housing of preweaned calves > 2 weeks of age		Group pen	170	0.27	0.007
		Single pen	18	0.18	
		Single and group pen	23	0.11	
		Missing	1	NE^e^	
Disinfection of pens of calves aged < 1 month		No	39	0.09	0.002
		Wet disinfectants	54	0.32	
		Dry disinfectants	77	0.27	
		Wet and dry desinfectants	41	0.28	
		Missing	3	0.29	
Frequency of milk feeding per day during first month of life		Two times	175	0.24	0.141
		Three times	22	0.21	
		Automatic milk feeder and other	15	0.42	
Antibiotics administered to calves with diarrhoea		No	87	0.17	0.019
		Yes	125	0.30	
Way of responding to questionnaire		Postal	127	0.24	0.667
		Phone	85	0.26	

^a^Within-herd mortality rate of calves in years 2017 to 2018 (per 100 calf-months)

^b^Estimated in bivariable negative binomial regression models including herd number of cows

^c^Dairy herd—at least 75% of cattle were of dairy breed; mixed herd – more than 25% of cattle were of beef breed

^d^Northeast Estonia: Ida-Viru, Lääne-Viru, Jõgeva, Järva county; Southeast Estonia: Tartu, Valga, Võru, Põlva county; Southwest Estonia: Pärnu, Viljandi, Saare county; Northwest Estonia: Harju, Rapla, Lääne, Hiiu county

^e^Not estimated due to small number of farms belonging to that category

^f^Other: calving on pasture and its combinations between other systems

Results from the final multivariable negative binomial regression model for calf mortality are presented in Table 2. The MR was higher in herds with larger proportion of abortions and stillbirths (p < 0.001). Management routines associated with higher calf MRs were prophylactic administration of vitamins to all calves (p = 0.012) compared with no administration and housing pre-weaned calves greater than 2 weeks of age in single pens only compared with housing in both single and group pens (p = 0.023). Larger herd size was associated with higher MR (p = 0.005), but the association was not linear. Calvings in multiple places compared with calvings restricted to group pens only was associated with lower MR (p = 0.008). Farmers that attended trainings two to three times during the last 4 years had increased risk of higher on-farm MRs of calves compared with those that attended trainings only once (incidence rate ratio (IRR) = 1.91, 95% CI 1.30; 2.81, p = 0.001). Farms located in the southeast part of the country had lower calf MRs (p = 0.013) compared with the northeast region (Table 2).

**Table 2:** Results of multivariable negative binomial regression analysis for risk factors associated with within-herd calf (aged 21–90 days) mortality rate in years 2017–2018 in 209 Estonian dairy herds

Variable	Categories	n	IRR^a^	95% confidence intervals	Category p-value	p-value
Herd size (centered) (number of cows^b^)			1.02	1.01; 1.03		0.005
Square term of herd size (centered)			0.9997	0.9995; 0.9999		0.001
Herd proportion of stillbirths (%)			1.09	1.04; 1.14		< 0.001
Herd proportion of abortions (%)			1.22	1.09; 1.36		< 0.001
Region^c^	Northeast	59	1			0.020
	Southeast	44	0.63	0.44; 0.90	0.013	
	Southwest	71	1.10	0.79; 1.53	0.573	
	Northwest	35	1.01	0.68; 1.52	0.946	
Farmer attending trainings within the last 4 years	Once	48	1			0.008
	2–3 times	107	1.91	1.30; 2.81	0.001	
	More than 3 times	31	1.34	0.83; 2.19	0.233	
	No	23	1.09	0.57; 2.08	0.792	
Prophylactic administration of vitamins to calves	No	70	1			0.041
	Sometimes	62	1.19	0.83; 1.69	0.337	
	Yes (to more than 95% of calves)	77	1.48	1.09; 2.01	0.012	
Housing of preweaned calves > 2 weeks of age	Group pen	168	1			0.020
	Single pen	18	1.17	0.67; 2.03	0.581	
	Single and group pen	23	0.51	0.32; 0.83	0.007	
Place of calving	Group pen	68	1			0.020
	Individual pen	41	0.83	0.59; 1.17	0.284	
	Tiestall	84	1.13	0.79; 1.61	0.510	
	Combined or other^d^	16	0.40	0.20; 0.78	0.008	

^a^Incidence rate ratio

^b^One unit change is 20 cows

^c^Northeast Estonia: Ida-Viru, Lääne-Viru, Jõgeva, Järva county; Southeast Estonia: Tartu, Valga, Võru, Põlva county; Southwest Estonia: Pärnu, Viljandi, Saare county; Northwest Estonia: Harju, Rapla, Lääne, Hiiu county

^d^Other: calving on pasture and its combinations between other systems

### Risk factors associated with cow mortality rate

Descriptive statistics and unconditional associations with cow MRs of all tested variables are presented in Additional files 3 and 4 for categorical and continuous variables, respectively. Variables that were considered to be candidates in the multivariable linear regression model for cow MR are presented in Tables 3 and 4 for continuous and categorical variables, respectively.

**Table 3:** Descriptive statistics and unconditional associations of continuous variables selected as candidates for multivariable model for within-herd cow mortality rate in years 2017–2018 in 212 Estonian dairy herds estimated in linear regression analysis

Variable	Median (Q1; Q3)	n	p-value^a^
Herd size (number of cows)	129.7 (53.7; 459.7)	212	< 0.001
Herd average milk yield per cow per year (kg)	8787 (6950; 9758)	212	0.002
Herd average milk fat/protein ratio	1.2 (1.2; 1.3)	212	0.001
Herd average age at first calving (days)	813.0 (764.3; 888.7)	212	0.024
Herd average interval from calving to insemination (days)	92.3 (79.6; 111.7)	204	0.061
Herd average number of inseminations per conception	1.9 (1.7; 2.2)	204	0.004
Herd average first insemination conception rate (%)	53.9 (47.2; 60.6)	204	0.003
Herd proportion of stillbirths (%)	7.5 (5.6; 9.5)	212	0.008
Herd proportion of abortions (%)	0.8 (0; 1.6)	212	0.022
Herd average number of lactations	2.4 (2.2; 2.7)	212	< 0.001
Herd average age at first insemination (days)	498.7 (457.3; 585.7)	187	0.123

^a^Estimated in bivariable linear regression models including herd number of cows to that category

**Table 4:** Descriptive statistics and unconditional associations of categorical variables selected as candidates for multivariable model for within-herd cow mortality rate in years 2017–2018 in 212 Estonian dairy herds estimated in linear regression analysis

Variable	Categories	n	Within-herd MR^a^	p-value^b^
Herd type^c^	Dairy herd	199	4.88	0.281
	Mixed herd	13	3.20	
Proportion of Holstein breed cows	< 90%	89	3.75	0.001
	> 90%	119	5.59	
	Missing	4	3.60	
Region^d^	Northeast	58	6.80	< 0.001
	Southeast	44	3.79	
	Southwest	72	3.88	
	Northwest	38	4.55	
Farmer attending trainings within the last 4 years	Once	49	4.10	0.018
	2–3 times	108	5.61	
	More than 3 times	31	4.36	
	No	24	2.97	
Farmer using consultancy service within the last 4 years	No	14	3.68	0.046
	Once	43	4.23	
	2–3 times	109	5.58	
	More than 3 times	46	3.75	
Handling animals	Farmer and/or family members	51	2.95	< 0.001
	Employees in addition to farmer/family	26	3.12	
	Employees only	135	5.79	
Veterinarian involved in detecting sick cows	No	168	4.36	0.026
	Yes	44	6.37	
Milking method	Pipeline milking/jug	81	3.71	0.033
	Milking parlour	91	5.41	
	Automatic milking system	35	5.48	
	Combined	5	5.65	
Housing system	Tie stall	76	3.75	0.022
	Free stall	130	5.30	
	Combined	6	6.72	
Grazing	No	83	5.79	< 0.001
	Yes	96	3.29	
	Only dry cows	33	6.57	
Bed surface material	Concrete	83	4.01	0.058
	Rubber	72	4.68	
	Mattress	35	6.58	
	Deep litter	17	4.90	
	Combined	5	5.96	
Analysing roughages in the laboratory	No	35	3.02	0.002
	Yes, not every year	29	4.13	
	Yes, every year	148	5.32	
Dry period feed ratio based on silage analysis	No	99	3.57	< 0.001
	Sometimes (in less than 50% of times)	13	4.75	
	Mostly (in more than 50% of times)	22	5.63	
	Always	76	6.16	
	Missing	2	NE^e^	
Frequency of hoof trimming	No	39	4.05	0.016
	Less than once a year	31	3.62	
	Once a year	60	4.24	
	≥ 2 times per year	82	5.96	
Digital dermatitis diagnosed in a herd	No or unaware	148	4.19	0.019
	Yes	62	6.18	
	Missing	2	NE^e^	
Place of calving	Group pen	69	5.94	0.005
	Individual pen	41	4.46	
	Tiestall	84	4.32	
	Combined or other^f^	16	3.25	
	Missing	2	NE^e^	
Use intramammary products at dry-off	No	23	3.84	0.045
	Sometimes (less than 50% of cows)	36	4.10	
	Often (50–75% of cows)	16	6.70	
	Mostly (75–95% of cows)	23	3.99	
	Always (more than 95% of cows)	114	5.07	
Way of responding to questionnaire	Postal	127	4.62	0.332
	Phone	85	5.02	

^a^Within-herd mortality rate of cows in years 2017 to 2018 (per 100 cow-years)

^b^Estimated in bivariable linear regression models including herd number of cows

^c^Dairy herd—at least 75% of cattle were of dairy breed; mixed herd – more than 25% of cattle were of beef breed

^d^Northeast Estonia: Ida-Viru, Lääne-Viru, Jõgeva, Järva county; Southeast Estonia: Tartu, Valga, Võru, Põlva county; Southwest Estonia: Pärnu, Viljandi, Saare county; Northwest Estonia: Harju, Rapla, Lääne, Hiiu county

^e^Not estimated due to small number of farms belonging to that category

^f^Other: calving on pasture and its combinations between other systems

According to the multivariable linear regression analysis, zero-grazing farms had higher cow MRs compared with farms that graze their cows (p = 0.034). In addition, grazing only dry cows was associated with significantly higher on-farm mortality hazard compared with farms that grazed all cows (the mean predicted on-farm MRs according to the linear regression model were 5.92, 95% CI 4.72; 7.11 and 3.34, 95% CI 2.57; 4.11 per 100 cow-years, respectively, p = 0.001). Farms that used only employed labour had higher cow MRs (MR = 5.17, 95% CI 4.61; 5.73) compared with farms that did not use employees (MR = 2.71, 95% CI 1.98; 3.45, p < 0.001). In general, farms that performed prophylactic hoof trimming (MR = 3.54, 95% CI 2.93; 4.16 in farms conducting hoof trimming once a year) had better cow survival rates compared with farms in which no prophylactic hoof trimming was applied within the last 4 years (MR = 5.86, 95% CI 4.58; 7.13, p < 0.001). Farms that housed their cows in free stalls had significantly lower MRs compared with farms that house in tie stalls (MR = 3.90, 95% CI 3.45; 4.35 and MR = 5.41, 95% CI 4.42; 6.40, respectively, p = 0.011). The negative effect of high stillbirth rate was present only in smaller farms (< 130 cows) (p < 0.001). Among larger herds (> 130 cows), the effect of the stillbirth rate was insignificant (p = 0.092). In addition, the highest risk of cow mortality occurred in herds located in northeast Estonia (MR = 5.95, 95% CI 5.12; 6.78, p < 0.001). Farms that had a higher average number of lactations experienced a lower mortality risk (p = 0.020) (Table 5). The final multivariable linear regression model adjusted R^2^ was 0.43.

**Table 5:** Results of multivariable linear regression analysis for risk factors for within-herd cow mortality rate over years 2017–2018 in 212 Estonian dairy herds

Variable	Categories	n	Coefficient^a^	95% Confidence Intervals	Category p-value	p-value
Handling animals	Farmer and/or family members	51	0			< 0.001
	Employees in addition to farmer/family	26	0.10	− 0.22; 0.42	0.543	
	Employees only	135	0.64	0.35; 0.93	< 0.001	
Housing system	Tie stall	76	0			0.008
	Free stall	130	-0.37	− 0.65; − 0.09	0.011	
	Combined	6	0.28	− 0.29; 0.85	0.337	
Grazing	No	83	0			0.006
	Yes	96	-0.37	− 0.72; − 0.03	0.034	
	Only dry cows	33	0.25	− 0.03; 0.52	0.076	
Frequency of prophylactic hoof trimming	No	39	0			0.002
	Less than once a year	31	-0.37	− 0.69; − 0.04	0.028	
	Once a year	60	-0.56	− 0.87; − 0.25	< 0.001	
	≥ 2 times per year	82	-0.31	− 0.67; 0.06	0.105	
Herd size x herd average proportion of stillbirths (SB)	Herds with < 130 cows and SB < 7.5%	59	0			< 0.001
	Herd with < 130 cows and SB ≥ 7.5%	47	0.57	0.29; 0.85	< 0.001	
	Herd with ≥ 130 cows and SB < 7.5%	47	0.29	− 0.05; 0.63	0.092	
	Herd with ≥ 130 cows and SB ≥ 7.5%	59	0.43	0.08; 0.78	0.016	
Herd average number of lactations		212	-0.30	− 0.55; − 0.05	0.020	0.020
Region^b^	Northeast	58	0			< 0.001
	Southeast	44	-0.75	− 1.01; − 0.48	< 0.001	
	Southwest	72	-0.41	− 0.66; − 0.16	0.001	
	Northwest	38	-0.46	− 0.75; − 0.18	0.002	
Intercept		212	3.08	2.30; 3.87	< 0.001	

^a^Square root transformation was made for the outcome variable „within-herd cow mortality rate “

^b^Northeast Estonia: Ida-Viru, Lääne-Viru, Jõgeva, Järva county; Southeast Estonia: Tartu, Valga, Võru, Põlva county; Southwest Estonia: Pärnu, Viljandi, Saare county; Northwest Estonia: Harju, Rapla, Lääne, Hiiu county

## Discussion

### Distribution of within-herd mortality rates

There was high between-herd variation in MRs of calves and cows, meaning that cattle health and welfare are highly dependent on herd-specific factors. Similar associations have been reported in previous studies [2, 19, 26]. Although many of the participating herds had no calf mortality events registered in their farms between years 2017 and 2018, it should be kept in mind that the calculated mortality estimate excluded the first 3 weeks of the lifetime of calves. Using registry data in this study, a reporting bias arises from unreported deaths of non-ear-tagged calves during early life [11, 28]. Farmers must ear-tag their calves within 20 days of life, and they have an additional 7 days after that to register their calves in the Animal Registry. This means that we were unable to get reliable estimates of calf MRs during the first 3 weeks of calves’ lifetime, known to bear the highest mortality risk among calves [11, 27, 28]. However, comparing the calf mortality with what has been reported in other studies for the same age group, the rates in the current study are similar to those reported in France, Germany and The Netherlands on animal level [27, 28, 32].

In the present study, the on-farm cow MRs might be somewhat underestimated because the exact first calving date was unknown, and the presented values should therefore be interpreted carefully. We decided to use the 25% quartile value of the age of first calving of Estonian dairy cows [30] when extracting the data frame for cow analyses to capture the majority of the first calvings in our analyses. In general, the estimated on-farm MRs are comparable to what has been reported in Swedish and Finnish studies [9, 19] and what was found previously at animal level in Estonia [11, 30].

### Risk factors associated with calf mortality rate

Several risk factors related to high calf MR were identified. The rate was higher in larger herds, which is consistent with previous studies that have reported a positive association between mortality and herd size [16, 18, 33]. Larger herds have different housing and management conditions compared with smaller farms, and individual attention to animals may be reduced [34]. The confounding effect of herd size was also present for some herd characteristics and management routines in our study. Unfortunately, we were not able to analyse risk factors separately for small and large-scale holdings due to limited sample size, but herd size was controlled for in the variable screening phase as well as in multivariable models to adjust for its effect as much as possible.

Herds with a higher proportion of abortions and stillbirths had also higher MRs. From previous studies we can conclude that perinatal mortality is a multifactorial problem including several animal-level risk factors related to both cows and the foetus/calf [35]. On a herd level, overall calving management (e.g., supervision of calvings, calving conditions including excessive prepartum body condition) plays an important role in addition to the farmer’s priorities in herd stillbirth rate [35–37]. Due to the aforementioned recording bias of non-ear-tagged calf mortality, the herd stillbirth rate includes at least some proportion of deaths of live-born calves. Therefore, due to positive association between herd stillbirth and calf MR, we may suppose that identical risk factors apply to both problems. Abortions often have an infectious aetiology [38], and some pathogens (e.g., bovine viral diarrhoea virus and bovine herpesvirus 1) are often pathogenic to young calves, causing bovine respiratory disease [39]. Respiratory and digestive tract diseases were the second most common cause of mortality of calves and the most common cause of death among pre-weaned calves [11], meaning that infectious diseases probably affect calf survival.

Combined housing conditions (calves confined in single and group pens, whereas the housing system might differ between calves or the system is changed at certain age) of pre-weaned calves greater than 2 weeks of age was associated with a lower calf MR on a herd level compared with housing calves in single pens only. By law, calves are allowed to be kept in individual pens up to 8 weeks of age, covering the majority of the preweaning period, if not otherwise specified by the veterinarian [40]. According to previous studies, housing calves in individual calf pens in early life is known to protect for mortality compared with group housing, where the risk of mortality tends to increase with group size [20, 21, 25, 33, 41]. In the current study, 71% of dairy farms housed their calves in individual calf pens during the first 2 weeks of life, whereas 80.2% housed them in group pens after that age. We might assume that farms using single pens for an extended period of time will have major problems with calf diseases and subsequently higher mortality. In addition, combined housing could be more flexible in moving calves between housing systems based on individual needs, thus improving health and survival rates.

A similar association was found for calving area in which the combined calving system (calving in multiple places) appeared to protect for calf mortality compared with having one single calving area/pen. The outcome in the risk factor model excluded calf mortality data from the first 3 weeks of life and, thus, it was surprising to identify this association. Interestingly, recent research from Sarjokari et al. [19] found that having group and single calving pens was associated with lower hazard for on-farm cow mortality compared with having a permanent calving pen. The authors explained the positive effect of a combined calving system by improved options to manage calvings, provide a peaceful environment and allow for supervision. This might also be essential regarding to the long-term positive effect of early calf health.

Prophylactic administration of vitamins to all calves was associated with higher calf MRs. Probably this practice is more common in herds with calfhood problems, aiming to compensate for poor health by boosting non-specific immunity. However, the effect of a single vitamin injection, which is the most common practice of providing vitamins to young calves in Estonian dairy farms, to the knowledge of the authors, might not yield significantly better health outcomes [42, 43]. However, Torsein et al. [5] found that fat-soluble vitamins might play an important role for calf health and possibly survival rates.

The current study identified a higher calf MR on farms in which farmers or farm managers attended more frequent trainings associated with cattle rearing, feeding and diseases. Again, this unexpected, apparently contradictory association might be found because farms with calfhood problems more actively look for advice and expertise. Nevertheless, according to Santman-Berends et al. [23], only a low proportion of farmers with high calf MRs claimed they had a lack of knowledge and felt they would need advice to solve their calf health-related problems. This means that farmers differ in their behaviour, and more research is needed to understand these patterns.

Differences in herd-level calf MR across regions occurred, where herds in the southeast region had the lowest MRs. The observed difference might be a result of distinct herd management factors, but further studies are needed to provide a more profound explanation.

Several questions were asked about colostrum management, rearing conditions and practices during the first weeks of calves’ life, but none of these factors appeared significant in the final models. Even so, it should be taken into consideration that we did not include deaths occurring during the first 3 weeks of life in the outcome, which possibly could explain these non-existing associations. According to the effect size of the variables in the model, it appears that calf housing and management (variables describing housing system, place of calving and administration of vitamins) as well as farmers’ activities and mind set (variable representing the farmer frequency of attending trainings) might be more influential than herd size in terms of calf health and survival.

### Risk factors associated with on-farm cow mortality rate

Zero grazing was associated with higher MRs in our study, and this was consistent with previous studies [9, 44, 45]. Although it remains unclear which ‘underlying factors’ actually cause a lower MR in cows spending more time in pasture [45], it is possible that the ability to express natural behaviour and regular exercise on a natural surface could be beneficial to cows’ health and welfare [46].

We identified that more frequent hoof trimming was associated with decreased MRs in cows. It has been confirmed that prophylactic hoof trimming reduces lameness [47]. Farmers reported the leg-and-claw disorder complex to be the second most common reason for unassisted death and euthanasia in Estonian dairy cows [11]. Likewise, it was the most common reason for on-farm cow mortality in Swedish dairy herds [48]. All actions aiming at lowering the incidence of leg and claw disorders may therefore represent valid measures in reducing cow MRs.

Farms where cows were managed by employees had higher MRs in agreement with findings by Weigel et al. [49]. The variable was strongly confounded by herd size, meaning that besides using external help, there might be other important factors explaining the association. It has been shown that larger herd size has contributed to less attention per cow and, thus, increased MRs [50]. Previous studies found a positive correlation between a cow-to-employee ratio and herd on-farm MRs [17, 49]. Intentionally, we did not inquire about this information in our survey to avoid obstructive questions and thus increasing the chance for a higher response rate. The type of labour used, and the cow-to-employee ratio should, however, be considered to allow for sound conclusions about the effect of employees versus farm owner’s presence on cattle health.

Higher herd average lactation number was associated with lower MRs, which makes sense since lower MRs and better longevity are related by context and both reflect the overall performance of the herd.

On-farm cow MRs were lower in loose-housing systems compared with tie stalls. Previous research has showed conflicting results, as some studies have found numerically lower cow MRs in tie stalls compared with free stall barns [9, 17], whereas the opposite also has been found [44]. These two farm systems differ in many aspects, but most probably there is a positive effect of movement, thermal conditions, feeding system and housing facilities in loose-housing systems that are more natural and supportive for cow health and welfare.

The herd-level MR of cows was higher in herds with increased percentage of stillbirths, but the effect was present only in smaller herds. This finding is in agreement with Shahid et al. [18] and Thomsen et al. [51], who argue that the association is a proxy for the farm-specific dairy cow management. On individual cow level, a positive association between having a high rate of stillborn calves and high on-farm cow mortality was found in many studies [18, 30, 48], demonstrating that the presence of stillbirth might also have a direct effect on cow health and result in death or euthanasia directly or via concurrent diseases.

Regional differences in cow MRs were confirmed, where herds in the northeast region appeared to have the highest MRs. The same association was also confirmed in our previous studies [11, 30], possibly reflecting regional differences in animal husbandry practices that were not captured in the present study.

### Limitations of the study

The overall response rate was good, and the responding herds in general represented Estonian dairy farms relatively well according to their locations. Due to the exclusion of the smallest herds with less than 20 cows and slight underrepresentation of herds with up to 50 cows, the study results should only be carefully extrapolated to smaller herds. Also, the responding herds may represent more interested farmers and therefore not reflect the population well in terms of the social factors. To our knowledge, this was also the first published insight into dairy cow housing conditions and management routines in Estonian dairy herds. Due to varying herd size and associated management routines, the analysis could have benefitted from stratified analyses across herd size categories and housing types; but, due to limited sample size, this was not possible. Another limitation of this study is that it lacks the ability of confirming causal associations due to the study design with a follow-up. This emphasises the need to use an alternative study design, on-farm assessment and measurements that would enable to have a clearer overview of the causal pathway of risk factors. The multivariable model of risk factors for mortality in cows explained 41% of the total variance of the MRs. This shows that there are many more factors that should be included in future studies to explain the complexity of the problem. Also, future research should focus on evaluating risk factors for calf and cow mortality in larger dairy herds as, due to intensification of the dairy production, the increase in herd size is inevitable.

## Conclusions

There was a high between-herd variability in on-farm MRs of ear-tagged calves and cattle older than 24 months. Registry mortality data could be used to detect problem herds and support guided allocation of resources to needed areas, thereby helping to improve general herd health. Herd size and housing conditions are important in determining calf mortality, but more focused insight is needed to provide clear recommendations. Our study results refer that providing good care and more natural living conditions might probably ensure better health and welfare of dairy calves and cows.

## Supplementary information

**Additional file 1.** Descriptive statistics and unconditional associations of categorical predictor variables estimated in negative binomial regression analysis for herd within-herd calf mortality rate in years 2017–2018 in 212 Estonian dairy herds.

**Additional file 2.** Descriptive statistics and unconditional associations of continuous predictor variables estimated in negative binomial regression analysis for within-herd calf mortality rate in years 2017–2018 in 212 Estonian dairy herds.

**Additional file 3.** Descriptive statistics and unconditional associations of categorical predictor variables estimated in linear regression analysis for herd within-herd cow mortality rate in years 2017–2018 in 212 Estonian dairy herds.

**Additional file 4.** Descriptive statistics and unconditional associations of continuous predictor variables estimated in linear regression analysis for herd within-herd cow mortality rate in years 2017–2018 in 212 Estonian dairy herds.

## Data Availability

The datasets analyzed during the current study are available from the corresponding author on reasonable request.
