# Supplementary material for: Herd-level risk factors for cow and calf on-farm mortality in Estonian dairy herds
Source: Acta Vet Scand. 2020 Mar 12;62:15. doi: 10.1186/s13028-020-0513-x (PMC7068997; doi:10.1186/s13028-020-0513-x)
Supplement: Supplementary file 1 — Additional file 1. Descriptive statistics and unconditional associations of categorical predictor variables estimated in negative binomial regression analysis for herd within-herd calf mortality rate in years 2017–2018 in 212 Estonian dairy herds. [file 13028_2020_513_MOESM1_ESM.docx]

| Additional file 1. Descriptive statistics and unconditional associations of categorical predictor variables estimated in negative binomial regression analysis for within-herd calf mortality rate in years 2017-2018 in 212 Estonian dairy herds | | | | |
| --- | --- | --- | --- | --- |
| Variable | Categories | n | Within-herd MR^a^ | ***P***-value^b^ |
| Herd type^c^ | Dairy herd | 199 | 0.26 | 0.035 |
|  | Mixed herd | 13 | 0.09 |  |
| Proportion of Holstein breed cows | <90% | 89 | 0.20 | 0.160 |
|  | >90% | 119 | 0.28 |  |
|  | Missing | 4 | 0.35 |  |
| Region^d^ | Northeast | 59 | 0.29 | 0.402 |
|  | Southeast | 44 | 0.19 |  |
|  | Southwest | 71 | 0.26 |  |
|  | Northwest | 38 | 0.22 |  |
| Purchase of new animals during years 2013-2016 | No | 128 | 0.24 | 0.999 |
|  | Yes | 84 | 0.26 |  |
| Major changes in farm during years 2013-2016 | No | 190 | 0.24 | 0.283 |
|  | Yes | 22 | 0.30 |  |
| Farmer attending trainings within the last four years | No | 24 | 0.14 | 0.002 |
|  | Once | 49 | 0.15 |  |
|  | 2-3 times | 108 | 0.33 |  |
|  | More than 3 times | 31 | 0.22 |  |
| Farmer using consultancy service within the last four years | No | 14 | 0.27 | 0.540 |
|  | Once | 43 | 0.21 |  |
|  | 2-3 times | 109 | 0.28 |  |
|  | More than 3 times | 46 | 0.19 |  |
| Veterinarian consulting in disease prevention issues | Yes | 152 | 0.24 | 0.776 |
|  | No | 56 | 0.25 |  |
|  | Missing | 4 | 0.30 |  |
| Place of calving | Group pen | 69 | 0.32 | 0.024 |
|  | Individual pen | 41 | 0.26 |  |
|  | Tie stall | 84 | 0.21 |  |
|  | Combined or other | 16 | 0.07 |  |
|  | Missing | 2 | NE^e^ |  |
| Proportion of assisted calvings | <10% | 80 | 0.24 | 0.543 |
|  | 11-30% | 70 | 0.28 |  |
|  | 31-50% | 39 | 0.20 |  |
|  | ≥51% | 23 | 0.24 |  |
| Separating newborn calf from the dam | Immediately | 70 | 0.29 | 0.245 |
|  | In an hour | 89 | 0.25 |  |
|  | In a day | 43 | 0.22 |  |
|  | Later than 24 hours | 10 | 0.10 |  |
| Time of feeding the first colostrum (hours after birth) | 0.5 h | 22 | 0.19 | 0.149 |
|  | 1 h | 87 | 0.23 |  |
|  | 2 h | 69 | 0.33 |  |
|  | 3 h | 17 | 0.19 |  |
|  | ≥ 4h | 12 | 0.14 |  |
|  | Calf drinks by itself | 3 | NE^e^ |  |
|  | As soon as possible | 2 | NE^e^ |  |
| Measuring colostrum quality | No | 132 | 0.21 | 0.591 |
|  | Sometimes | 40 | 0.32 |  |
|  | Always (in more than 95% of calves) | 40 | 0.30 |  |
| Feeding colostrum via oesophageal tube to calves | No | 152 | 0.22 | 0.626 |
|  | Yes | 60 | 0.31 |  |
| Disinfecting navel cord | No | 68 | 0.21 | 0.070 |
|  | Sometimes | 51 | 0.17 |  |
|  | Always (in more than 95% of calves) | 93 | 0.32 |  |
| Prophylactic administration of antibiotics to calves | No | 179 | 0.24 | 0.721 |
|  | Yes | 33 | 0.27 |  |
| Prophylactic administration of vitamins to calves | No | 64 | 0.22 | 0.101 |
|  | Sometimes | 70 | 0.21 |  |
|  | Yes (to more than 95% of calves) | 78 | 0.31 |  |
| Barn type of calves aged <2 weeks | Cold barn | 41 | 0.30 | 0.760 |
|  | Warm barn | 168 | 0.23 |  |
|  | Other | 3 | 0.19 |  |
| Housing of calves aged <2 weeks | Single pen | 150 | 0.22 | 0.031 |
|  | Group pen | 25 | 0.22 |  |
|  | Both | 37 | 0.39 |  |
| Access to outside area for <3 months old calves | No | 168 | 0.26 | 0.344 |
|  | Yes | 43 | 0.18 |  |
|  | Missing | 1 | NE^e^ |  |
| Housing of preweaned calves >2 weeks of age | Single pen | 18 | 0.18 | 0.007 |
|  | Group pen | 170 | 0.27 |  |
|  | Single and group pen | 23 | 0.11 |  |
|  | Missing | 1 | NE^e^ |  |
| Disinfection of pens of calves aged <1 month | No | 39 | 0.09 | 0.002 |
|  | Wet disinfectants | 54 | 0.32 |  |
|  | Dry disinfectants | 77 | 0.27 |  |
|  | Wet and dry desinfectants | 41 | 0.28 |  |
|  | Missing | 3 | 0.29 |  |
| Frequency of milk feeding per day during first month of life | Two times | 175 | 0.24 | 0.141 |
|  | Three times | 22 | 0.21 |  |
|  | Automatic milk feeder and other | 15 | 0.42 |  |
| Feeding calves milk with antibiotic residues | No | 174 | 0.24 | 0.352 |
|  | Yes | 38 | 0.30 |  |
| Feeding calves milk with high somatic cell count | No | 156 | 0.25 | 0.922 |
|  | Yes | 56 | 0.23 |  |
| Age of calf at disbudding | No disbudding | 52 | 0.18 | 0.674 |
|  | <1 month | 114 | 0.26 |  |
|  | Older than 1 month | 46 | 0.29 |  |
| Age since roughages are provided to calves | First week of life | 128 | 0.24 | 0.457 |
|  | First month of life | 75 | 0.26 |  |
|  | After first month of life | 9 | 0.17 |  |
| Age since water is provided to calves | First week of life | 139 | 0.28 | 0.319 |
|  | After first week of life | 73 | 0.19 |  |
|  | Missing | 2 | NE^e^ |  |
| Separating sick calves | No | 41 | 0.31 | 0.235 |
|  | Sometimes | 83 | 0.27 |  |
|  | Always (in more than 95% of cases) | 75 | 0.20 |  |
|  | Calves are housed in individual pens | 13 | 0.14 |  |
| Milk fed to calves with diarrhea | No | 126 | 0.25 | 0.555 |
|  | Yes | 86 | 0.25 |  |
| Electrolytes provided to calves with diarrhoea | No | 33 | 0.21 | 0.993 |
|  | Yes | 179 | 0.25 |  |
| Antibiotics administered to calves with diarrhoea | No | 87 | 0.17 | 0.019 |
|  | Yes | 125 | 0.30 |  |
| Way of responding to questionnarie | Postal | 127 | 0.24 | 0.667 |
|  | Phone | 85 | 0.26 |  |
| ^a^Within-herd mortality rate of calves in years 2017 to 2018 (per 100 calf-months) | | | | |
| ^b^Estimated in bivariable negative binomial regression models including herd number of cows | | | | |
| ^c^Dairy herd – at least 75% of cattle were of dairy breed; mixed herd – more than 25% of cattle were of beef breed | | | | |
| ^d^Northeast Estonia: Ida-Viru, Lääne-Viru, Jõgeva, Järva county; Southeast Estonia: Tartu, Valga, Võru, Põlva county; Southwest Estonia: Pärnu, Viljandi, Saare county; Northwest Estonia: Harju, Rapla, Lääne, Hiiu county | | | | |
| ^e^Not estimated due to small number of farms belonging to that category | | | | |
