# Supplementary material for: Herd-level risk factors for cow and calf on-farm mortality in Estonian dairy herds
Source: Acta Vet Scand. 2020 Mar 12;62:15. doi: 10.1186/s13028-020-0513-x (PMC7068997; doi:10.1186/s13028-020-0513-x)
Supplement: Supplementary file 2 — Additional file 2. Descriptive statistics and unconditional associations of continuous predictor variables estimated in negative binomial regression analysis for within-herd calf mortality rate in years 2017–2018 in 212 Estonian dairy herds. [file 13028_2020_513_MOESM2_ESM.docx]

| Additional file 2. Descriptive statistics and unconditional associations of continuous predictor variables estimated in negative binomial regression analysis for within-herd calf mortality rate in years 2017-2018 in 212 Estonian dairy herds | | | | | |
| --- | --- | --- | --- | --- | --- |
| Variable | Median | Quartiles | Missing obs (n) | IRR^a^ | ***P***-value^b^ |
| Herd average number of cows | 129.7 | 53.7; 459.7 | 0 | 1.0005 | 0.015 |
| Herd average milk yield per cow per year (kg) | 8787 | 6950; 9758 | 0 | 1.00007 | 0.132 |
| Herd proportion of stillbirths (%) | 7.50 | 5.61; 9.47 | 0 | 1.053 | 0.018 |
| Herd proportion of abortions (%) | 0.80 | 0; 1.56 | 0 | 1.259 | <0.001 |
| Farmer´s number of years worked | 24 | 15; 30 | 2 | 1.0004 | 0.949 |
| ^a^Incidence rate ratio |  |  |  |  |  |
| ^b^Estimated in bivariable negative binomial regression models including herd size | | | | | |
