# Supplementary material for: Herd-level risk factors for cow and calf on-farm mortality in Estonian dairy herds
Source: Acta Vet Scand. 2020 Mar 12;62:15. doi: 10.1186/s13028-020-0513-x (PMC7068997; doi:10.1186/s13028-020-0513-x)
Supplement: Supplementary file 3 — Additional file 3. Descriptive statistics and unconditional associations of categorical predictor variables estimated in linear regression analysis for herd within-herd cow mortality rate in years 2017–2018 in 212 Estonian dairy herds. [file 13028_2020_513_MOESM3_ESM.docx]

| Additional file 3. Descriptive statistics and unconditional associations of categorical predictor variables estimated in linear regression analysis for within-herd cow mortality rate in years 2017-2018 in 212 Estonian dairy herds | | | | | |
| --- | --- | --- | --- | --- | --- |
| Variable | Categories | n | Within-herd MR^a^ | ***P***-value^b^ | |
| Herd type^c^ | Dairy herd | 199 | 4.88 | 0.281 | |
|  | Mixed herd | 13 | 3.20 |  | |
| Proportion of Holstein breed cows | <90% | 89 | 3.75 | 0.001 | |
|  | >90% | 119 | 5.59 |  | |
|  | Missing | 4 | 3.60 |  | |
| Region^d^ | Northeast | 58 | 6.80 | <0.001 | |
|  | Southeast | 44 | 3.79 |  | |
|  | Southwest | 72 | 3.88 |  | |
|  | Northwest | 38 | 4.55 |  | |
| Purchase of new animals during years 2013-2016 | No | 128 | 4.90 | 0.207 | |
|  | Yes | 84 | 4.59 |  | |
| Mayor changes in farm during years 2013-2016 | No | 190 | 4.70 | 0.280 | |
|  | Yes | 22 | 5.50 |  | |
| Farmer attending trainings within the last four years | No | 24 | 2.97 | 0.018 | |
|  | Once | 49 | 4.10 |  | |
|  | 2-3 times | 108 | 5.61 |  | |
|  | More than 3 times | 31 | 4.36 |  | |
| Farmer using consultancy service within the last four years | No | 14 | 3.68 | 0.046 | |
|  | Once | 43 | 4.23 |  | |
|  | 2-3 times | 109 | 5.58 |  | |
|  | More than 3 times | 46 | 3.75 |  | |
| Handling animals | Farmer and/or family members | 51 | 2.95 | <0.001 | |
|  | Employees in addition to farmer/family | 26 | 3.12 |  | |
|  | Employees only | 135 | 5.79 |  | |
| Veterinarian involved in detecting sick cows | No | 168 | 4.36 | 0.026 | |
|  | Yes | 44 | 6.37 |  | |
| Veterinarian consulting in disease prevention issues | Yes | 152 | 4.73 | 0.790 | |
|  | No | 56 | 4.90 |  | |
|  | Missing | 4 | 4.97 |  | |
| Milking method | Pipeline milking/jug | 81 | 3.71 | 0.028 | |
|  | Milking parlour | 91 | 5.41 |  | |
|  | Automatic milking system | 35 | 5.48 |  | |
|  | Combined | 5 | 5.65 |  | |
| Housing system | Tie stall | 76 | 3.75 | 0.022 | |
|  | Free stall | 130 | 5.30 |  | |
|  | Combined | 6 | 6.72 |  | |
| Grazing | No | 83 | 5.79 | <0.001 | |
|  | Yes | 96 | 3.29 |  | |
|  | Only dry cows | 33 | 6.57 |  | |
| Bed surface material | Concrete | 83 | 4.01 | 0.058 | |
|  | Rubber | 72 | 4.68 |  | |
|  | Mattress | 35 | 6.58 |  | |
|  | Deep litter | 17 | 4.90 |  | |
|  | Combined | 5 | 5.96 |  | |
| Bedding material | None | 51 | 5.35 | 0.318 | |
|  | Sawdust | 43 | 5.15 |  | |
|  | Straw/hay | 69 | 4.28 |  | |
|  | Combined | 31 | 3.86 |  | |
|  | Other | 18 | 5.78 |  | |
| Floor of the walking alleys | Concrete ≥ 75% | 173 | 4.58 | 0.501 | |
|  | Rubber >25% | 27 | 4.97 |  | |
|  | Missing | 12 | 7.31 |  | |
| Access to roughages | ≥ 20 hours/day | 19 | 3.89 | 0.220 | |
|  | < 20 hours/day | 193 | 4.87 |  | |
| Analysing roughages in the laboratory | No | 35 | 3.02 | 0.002 | |
|  | Yes, not every year | 29 | 4.13 |  | |
|  | Yes, every year | 148 | 5.32 |  | |
| Number of feed groups | More than one for lactating cows | 92 | 5.22 | 0.849 | |
|  | Same for lactating and dry cows | 15 | 4.57 |  | |
|  | Separate for lactating cows and dry cows | 81 | 4.42 |  | |
|  | Concentrates based on production | 14 | 5.28 |  | |
|  | Missing | 10 | 3.29 |  | |
| Feeding concentrates precalving | No concentrates | 34 | 4.36 | 0.425 | |
|  | Less than a week | 13 | 3.03 |  | |
|  | 2-3 weeks | 121 | 4.93 |  | |
|  | >3 weeks | 31 | 4.59 |  | |
|  | Missing | 13 | 6.67 |  | |
| Feeding dry period minerals to cows and heifers precalving | Yes | 198 | 4.73 | 0.843 | |
|  | Sometimes or no | 14 | 5.44 |  | |
| Dry period feed ratio is based on silage analysis | No | 99 | 3.57 | <0.001 | |
|  | Sometimes (in less than 50% of times) | 13 | 4.75 |  | |
|  | Mostly (in more than 50% of times) | 22 | 5.63 |  | |
|  | Always | 76 | 6.16 |  | |
|  | Missing | 2 | NE^e^ |  | |
| Use of ketosis prophylactic products | No | 66 | 4.71 | 0.789 | |
|  | Energy drinks including propylene glycol | 86 | 4.41 |  | |
|  | Rumen boluses | 19 | 5.79 |  | |
|  | Combination of products and other products | 40 | 5.18 |  | |
|  | Missing | 1 | NE^e^ |  | |
| Frequency of body condition scoring | No scoring | 102 | 4.98 | 0.496 | |
|  | Less than once a year | 17 | 5.50 |  | |
|  | 1-3 times per year | 46 | 3.99 |  | |
|  | At least 4 times a year | 46 | 4.84 |  | |
|  | Missing | 1 | NE^e^ |  | |
| Frequency of prophylactic hooftrimming | No | 39 | 4.05 | 0.016 | |
|  | Less than once a year | 31 | 3.62 |  | |
|  | Once a year | 60 | 4.24 |  | |
|  | ≥2 times per year | 82 | 5.96 |  | |
| Trimming lame cows | No | 31 | 4.78 | 0.917 | |
|  | Sometimes (less than 50% of lame cows) | 41 | 4.54 |  | |
|  | Often (50-75% of lame cows) | 28 | 5.04 |  | |
|  | Mostly (75-95% of lame cows) | 42 | 4.94 |  | |
|  | Always (more than 95% of lame cows) | 70 | 4.72 |  | |
| Digital dermatitis diagnosed in a herd | No or unaware | 148 | 4.19 | 0.019 | |
|  | Yes | 62 | 6.18 |  | |
|  | Missing | 2 | NE^e^ |  | |
| Use of hoof baths | No | 174 | 4.49 | 0.236 | |
|  | Yes | 38 | 6.09 |  | |
| Place of calving | Group pen | 69 | 5.94 | 0.005 | |
|  | Individual pen | 41 | 4.46 |  | |
|  | Tie stall | 84 | 4.32 |  | |
|  | Combined or other | 16 | 3.25 |  | |
|  | Missing | 2 | NE^e^ |  | |
| Proportion of assisted calvings | <10% | 80 | 4.30 | 0.264 | |
|  | 11-30% | 70 | 5.29 |  | |
|  | 31-50% | 39 | 4.72 |  | |
|  | ≥51% | 23 | 4.98 |  | |
| Check uterus in majority (>90%) of post-partum cows | No | 130 | 4.76 | 0.489 | |
|  | Yes | 82 | 4.82 |  | |
| Measure body temperature in majority (>90%) of post-partum cows | No | 161 | 4.62 | 0.928 | |
|  | Yes | 51 | 5.26 |  | |
| Test ketosis in majority (>90%) of post-partum cows | No | 170 | 4.66 | 0.647 | |
|  | Yes | 42 | 5.26 |  | |
| Check lameness in majority (>90%) of post-partum cows | No | 210 | 4.76 | 0.514 | |
|  | Yes | 2 | NE^e^ |  | |
| Use intramammary products at dry-off | No | 23 | 3.84 | 0.045 | |
|  | Sometimes (less than 50% of cows) | 36 | 4.10 |  | |
|  | Often (50-75% of cows) | 16 | 6.70 |  | |
|  | Mostly (75-95% of cows) | 23 | 3.99 |  | |
|  | Always (more than 95% of cows) | 114 | 5.07 |  | |
| Way of responding to questionnarie | Postal | 127 | 4.62 | 0.332 | |
|  | Phone | 85 | 5.02 |  | |
| ^a^Within-herd mortality rate of cows in years 2015 to 2017 (per 100 cow-years) | | | | |  |
| ^b^Assessed in linear regression models including herd size | | | | |  |
| ^c^Dairy herd – at least 75% of cattle were of dairy breed; mixed herd – more than 25% of cattle were of beef breed | | | | |  |
| ^d^Northeast Estonia: Ida-Viru, Lääne-Viru, Jõgeva, Järva county; Southeast Estonia: Tartu, Valga, Võru, Põlva county; Southwest Estonia: Pärnu, Viljandi, Saare county; Northwest Estonia: Harju, Rapla, Lääne, Hiiu county | | | | |  |
| ^e^Estimates not estimable due to small number of farms belonging to that category | | | | |  |
