# Supplementary material for: Herd-level risk factors for cow and calf on-farm mortality in Estonian dairy herds
Source: Acta Vet Scand. 2020 Mar 12;62:15. doi: 10.1186/s13028-020-0513-x (PMC7068997; doi:10.1186/s13028-020-0513-x)
Supplement: Supplementary file 4 — Additional file 4. Descriptive statistics and unconditional associations of continuous predictor variables estimated in linear regression analysis for herd within-herd cow mortality rate in years 2017–2018 in 212 Estonian dairy herds. [file 13028_2020_513_MOESM4_ESM.docx]

| Additional file 4. Descriptive statistics and unconditional associations of continuous predictor variables estimated in linear regression analysis for within-herd cow mortality rate in years 2017-2018 in 212 Estonian dairy herds | | | | | |
| --- | --- | --- | --- | --- | --- |
| Variable | Median | Quartiles | Missing observations (n) | Coefficient^a^ | ***P***-value^b^ |
| Herd average number of cows | 129 | 53.67; 459.67 | 0 | 0.0007 | <0.001 |
| Herd average milk yield per cow per year (kg) | 8787 | 6949.67; 9758.00 | 0 | 0.0001 | 0.002 |
| Herd average milk fat/protein ratio | 1.21 | 1.15; 1.26 | 0 | -2.311 | 0.001 |
| Herd average milk somatic cell count (*1000/ml) | 283 | 217.17; 363.33 | 0 | -0.0004 | 0.304 |
| Herd average milk urea level (mg/l) | 239 | 219.50; 254.83 | 0 | 0.0004 | 0.841 |
| Herd average age at first calving (days) | 813 | 764.33; 888.67 | 0 | -0.001 | 0.024 |
| Herd average calving interval (days) | 414 | 401.67; 436.33 | 0 | -0.002 | 0.411 |
| Herd average length of dry period (days) | 66 | 59.67; 72.83 | 0 | -0.002 | 0.676 |
| Herd average interval from calving to insemination (days) | 92 | 79.58; 111.67 | 8 | -0.003 | 0.061 |
| Herd average calving to conception interval (days) | 135 | 123.00; 158.67 | 7 | -0.002 | 0.266 |
| Herd average number of inseminations per conception | 1.9 | 1.65; 2.19 | 8 | 0.416 | 0.004 |
| Herd average first insemination conception rate (%) | 53.9 | 47.25; 60.63 | 8 | -0.013 | 0.003 |
| Herd proportion of stillbirths (%) | 7.5 | 5.61; 9.47 | 0 | 0.043 | 0.008 |
| Herd proportion of abortions (%) | 0.8 | 0; 1.56 | 0 | 0.107 | 0.022 |
| Herd average number of lactations | 2.4 | 2.23; 2.71 | 0 | -0.567 | <0.001 |
| Herd average age at first insemination (days) | 499 | 457.33; 585.67 | 25 | -0.0009 | 0.123 |
| Farmer´s number of years worked | 24 | 15.00; 30.00 | 2 | -0.004 | 0.487 |
| ^a^Square root transformation was made for the outcome variable „within-herd cow mortality rate“ | | | | | |
| ^b^Assessed in bivariable linear regression models including herd size | | | | | |
